# Concise Polygenic Models for Cancer-Specific Identification of Drug-Sensitive Tumors from Their Multi-Omics Profiles

**DOI:** 10.3390/biom10060963

**Published:** 2020-06-26

**Authors:** Stefan Naulaerts, Michael P. Menden, Pedro J. Ballester

**Affiliations:** 1 Cancer Research Center of Marseille, INSERM U1068, F-13009 Marseille, France; stefan.naulaerts@bru.licr.org; 2 Institut Paoli-Calmettes, F-13009 Marseille, France; 3 Aix-Marseille Université, F-13284 Marseille, France; 4 CNRS UMR7258, F-13009 Marseille, France; 5 Ludwig Institute for Cancer Research, de Duve Institute, Université catholique de Louvain, 1200 Brussels, Belgium; 6 Institute of Computational Biology, Helmholtz Zentrum München—German Research Center for Environmental Health, 85764 Neuherberg, Germany; michael.menden@helmholtz-muenchen.de; 7 Department of Biology, Ludwig-Maximilians University Munich, 82152 Planegg-Martinsried, Germany; 8 German Centre for Diabetes Research (DZD e.V.), 85764 Neuherberg, Germany

**Keywords:** cancer pharmacogenomics, machine learning, feature selection, model interpretability, drug resistance

## Abstract

In silico models to predict which tumors will respond to a given drug are necessary for Precision Oncology. However, predictive models are only available for a handful of cases (each case being a given drug acting on tumors of a specific cancer type). A way to generate predictive models for the remaining cases is with suitable machine learning algorithms that are yet to be applied to existing in vitro pharmacogenomics datasets. Here, we apply XGBoost integrated with a stringent feature selection approach, which is an algorithm that is advantageous for these high-dimensional problems. Thus, we identified and validated 118 predictive models for 62 drugs across five cancer types by exploiting four molecular profiles (sequence mutations, copy-number alterations, gene expression, and DNA methylation). Predictive models were found in each cancer type and with every molecular profile. On average, no omics profile or cancer type obtained models with higher predictive accuracy than the rest. However, within a given cancer type, some molecular profiles were overrepresented among predictive models. For instance, CNA profiles were predictive in breast invasive carcinoma (BRCA) cell lines, but not in small cell lung cancer (SCLC) cell lines where gene expression (GEX) and DNA methylation profiles were the most predictive. Lastly, we identified the best XGBoost model per cancer type and analyzed their selected features. For each model, some of the genes in the selected list had already been found to be individually linked to the response to that drug, providing additional evidence of the usefulness of these models and the merits of the feature selection scheme.

## 1. Background

Large-scale cancer in vitro pharmacogenomics databases have been generated over the last six years. The most well-known are the Cancer Cell Line Encyclopedia (CCLE)-based project [1], Cancer Therapeutics Response Portal (CTRP) [2], and the Genomics of Drug Sensitivity in Cancer (GDSC) [3,4,5], which provide a deep molecular characterization (mutations, copy-number alterations, DNA methylations, and gene expression levels) of large panels of cell lines prior to being tested with hundreds of drugs. Those datasets have been growing ever since; for example, the latest GDSC study^5^ employed 265 drugs characterized in 1001 cell lines, with 990 cell line drug responses being readily available. CCLE/CTRP and GDSC have expanded their cell line panels by more than 16-fold compared to the pioneering NCI-60 panel [6], enabling the study of over 30 cancer types in detail, albeit restricting their screening effort to a smaller set of clinical and pre-clinical compounds. These screening efforts describe the heterogeneous landscape of cancer and explore pharmacological responses to drug treatments, which ultimately enables Precision Oncology.

The resulting pharmacogenomics databases have in turn spurred the generation of a range of computational models for drug response prediction in cancer cell lines [7,8,9]. These predictive models have been shown to be accurate in a few binomials drug-cancer type [5,10,11]. Building these models constitutes a strongly high-dimensional problem and, as such, it should employ some form of feature selection. It is indeed error-prone to directly relate many thousands of molecular features to a far smaller number of drug-treated cell lines (here data instances). Consequently, some studies [5,12] have followed a knowledge-based approach to select likely relevant subsets of features: e.g., restricting to those genes in the cancer gene census set [13], or to restrict to mutations also observed in TCGA tumor profiles [5]. Purely data-driven feature selection has also been employed to mitigate this form of overfitting, e.g., studies using feature selection embedded in Random Forest (RF) [14,15,16,17]. However, even such learning algorithms tend to overfit training data due to their model complexity [18]. An overfitted model predicts training data much better than test data, which we have observed when using learning algorithms such as RF [15,19] or XGBoost [20,21]. An attractive way to reduce model complexity is reducing the number of input features, but if such a reduction is too large, then it will cause model underfitting (i.e., the model not being sufficiently complex for the data). To tackle these potential issues, we will later introduce an adaptive and data-driven feature selection scheme to build models of optimal complexity.

The DREAM 7 challenge [11] attempted to establish which families of algorithms performed best to predict drug sensitivity for a panel of 53 breast cancer cell lines. Although no substantially superior algorithm was found, it was observed that the top-performing models were those accounting for nonlinearities. In that study, the best performing model was a Bayesian multitask multiple kernel learning approach, while the runner-up was built upon an underlying RF model. These studies are still scarce in that accurate predictors are still needed for many drugs across many cancer types [22] and comparative benchmarking is even rarer.

One way to identify accurate predictors for orphan drug–cancer type pairs is to apply new machine learning algorithms, preferably those integrated with feature selection. XGBoost (XGB for short) [23] has frequently been part of winner solutions in Kaggle (www.kaggle.com) competitions [23]. Similar to the well-established RF algorithm, XGB is an ensemble tree-based model built on CART (Classification And Regression Trees). However, XGB adds gradient boosting and is known to require substantially less training time than RF on most problem instances [24]. XGB has also been found to provide predictions almost as good as those of deep neural nets on the related problem of QSAR (Quantitative Structure–Activity Relationship) modeling, while running in less than one-third of its wall-clock time [24]. In addition, XGB has excelled at predicting drug–target binding affinities [25], where it outperformed widely used regularized least-squares techniques on the studied datasets. As its constituent tree models ignore by construction irrelevant features, XGB has embedded feature selection, which makes it an algorithm suitable to analyze high-dimensional data to some extent. Due to these qualities, some aspects of the application of the off-the-shelf XGBoost algorithm have been investigated using GDSC datasets [26].

In this study, we apply new XGB algorithms to GDSC data to identify novel cancer-specific models predictive of drug response. In addition to standard XGB, we introduce and apply an XGB variant integrating an adaptive feature selection scheme. This new scheme is called Optimal Model Complexity (OMC), and it complements the embedded feature selection of XGB by searching for a much smaller subset of features, which often results in less overfitting and thus more predictive models. An additional advantage of the OMC strategy is that a small set of features allows better interpretability and less resource-intensive application to other tumor samples than models employing all the available features (note that other model interpretability techniques from the area of Explainable Artificial Intelligence, XAI [27], could alternatively be applied to this problem). Here, we show the complementarity of both XGB algorithms across the drug–cancer type binomials for which new predictors were found. Furthermore, we identify the most predictive molecular profile for each of these binomials. XGB-OMC predictors exploiting gene expression or DNA methylation profiles are particularly innovative, as these profiles have not been previously analyzed with nonlinear regression algorithms that are able to find a concise subset of predictive features.

## 2. Data Description

We defined each case as a drug and cancer type pair, where cell lines can be described by four omics profiles. In terms of genomic profiles, we analyzed Single-Nucleotide Variants (SNV) and CNA data. Both were encoded as binary features, each feature indicating whether a mutation was detected in the gene or if a chromosomal region had copy-number alterations, respectively. On the other hand, gene expression intensities (RMA normalized mapped to gene) and methylation ß-values of the CpG islands were used as real-valued features. Regarding drug sensitivity data, we found 990 cell lines with 212,774 log IC50 values for 265 drugs in 30 cancer types in the GDSC (49,576 measurements did not have an associated cancer type and were subsequently discarded). Once we restricted the dataset to the cancer types with at least 45 cell lines, 3167 unique triplets of drug, cancer type, and tumor profile type remained. Each of these triplets had between 45 and 62 fully profiled data instances, comprising 219 drugs across five cancer types (BRCA: breast invasive carcinoma, COAD/READ: colorectal adenocarcinoma/rectum adenocarcinoma, LUAD: lung adenocarcinoma, SCLC: small cell lung cancer, and SKCM: skin cutaneous melanoma). Drug response is quantified with the concentration required to reduce cell viability by half, i.e., the IC50 value. The log IC50 of an instance is the dependent continuous variable for which regression models will be built. Further description of the data and their pre-processing is available in the Methods section.

**Figure 1:**
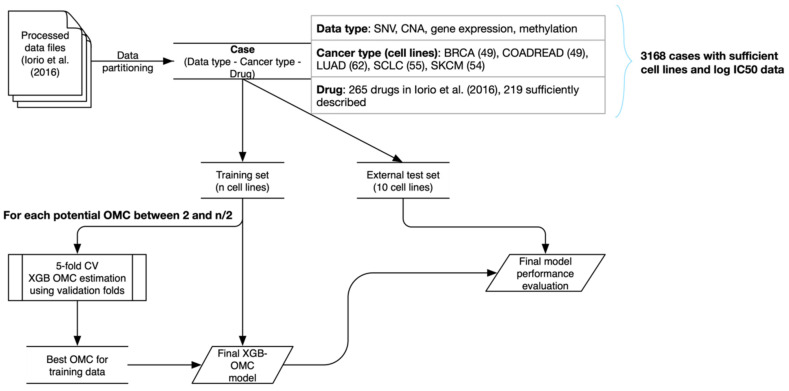
Schematic outline of the procedure to determine the Optimal Model Complexity (OMC) for each case and evaluating the resulting models. Data pre-processed in the latest Genomics of Drug Sensitivity in Cancer (GDSC) study [5] is partitioned into unique cases and then split into training and test sets. A five-fold cross-validation using stratified folds is used to establish which are the top k features (where k takes all the integer values between 2 and *n*/2, with *n* being the number of training cell lines), leading to the most predictive XGB regression model over the five cross-validation runs. Once the optimal k (k_opt) has been determined, the top k_opt features on the entire training set are identified. This is repeated for each case, giving rise to the predictive models discovered and analyzed in this study.

To assess the predictive power of the generated models (Figure 1), we split the cell lines of each case into a training set (used also for model selection via cross-validation) and an external test set. Hence, there is no overlap between any training and test sets. Every test set contained 10 cell lines and was stratified to ensure that it is representative of drug responses for the considered case. In a nutshell, the stratification consists of selecting cell lines at equidistant log IC50 ranks, so that both training and test sets have the same log IC50 range (see Methods subsection *“Generation of non-overlapping training and test sets”*). The number of training cell lines, *n*, varies from case to case. For the training set of each case, OMC considered the top k features, with k ranging from 2 to *n*/2 and *n* being the number of cell lines in the training set of the considered case. This was done in order to generate a sequence of (*n*/2) − 1 nested feature subsets, each leading to a different training set using the same cell lines. We set *n*/2 as the highest model complexity to have at least two training data instances per feature (i.e., two training cell lines per feature as a heuristic to identify models that only require a concise list of features). The OMC was chosen as the top k features resulting in the best median performance over the five folds. The full list of features for the case is also used to build a baseline model with all features and running them on the same training set as the OMC strategy.

In order to select the OMC, features need to be ranked first. We used the *p*-values obtained from univariate statistical tests to correlate between each feature and the corresponding labels (log IC50 values). For a given case and complexity, *p*-values between each feature and drug response were calculated using only the training folds (i.e., excluding the validation fold) within the 5-fold stratified cross-validation (see Figure 1). Note that the test set is not used at any step of this training process; in particular, the determination of the OMC uses the training set exclusively. For discrete features (SNV, CNA) and continuous features (gene expression intensities, methylation β-values), the *p*-values of the Wilcoxon rank-sum test and the Spearman rank-correlation test were used, respectively. We opted to use univariate filter methods to avoid long computational times, making assumptions or biasing predictive performance, as previously reported in the literature [28] (this study found univariate filters to be among the top-performing feature selection algorithms in a related high-dimensional problem).

We estimated the OMC using a five-fold stratified cross-validation for model selection. To this end, log IC50 values were randomly distributed to one of five folds, yet their ranking within a fold was maintained from small to large in order to represent a fold representative of the test set and the full drug response range. Sizes of validation set folds varied between 7 and 11 cell lines, which is comparable to the size of the external test set. Therefore, we expected the validation folds to provide a good representation of the behavior of the model in the test set. To promote that the OMC generalizes well, we required that the minimum Spearman correlation (R_s_) of all five validation folds exceeded an R_s_ of 0.25 (this is a cut-off previously used to model these datasets [5]); if this minimum performance level is not reached, that OMC is no longer considered. Once the OMC is determined, *p*-values are calculated using the entire training set, and only the top k_opt features are employed to train the XGB-OMC model, which is applied to predict drug sensitivities in the test set (see Figure 1).

To summarize, we obtain combinations of a drug, cancer type, and omics level. These datasets were each separately analyzed. If we find complexities generalizing well across all five folds, we keep this complexity and use it to build the final model. The final model is trained on the full training set and uses only the top-k elements that best correlate with the drug’s response.

## 3. Results and Discussion

### 3.1. Building Predictive Cancer-Specific Models

For a model to be regarded as potentially predictive, there needs to be sufficient correlation between its predictions and the true observed log IC50 values. Thus, we required a minimum R_s_ of 0.25 in every validation fold for a model to be considered as potentially predictive. For a potentially predictive model to be confirmed as predictive, we also required that the model needed to have an R_s_ value greater than 0.25 in the test set. By eliminating those with performance below this test set threshold, the number of models was reduced from 268 to 77 (28.73%). The 77 predictive models cover 62 unique drugs across all four data types in the five cancer types with sufficient cell lines. These models are specified in the Supplementary Materials (Supplementary file 1).

**Figure 2:**
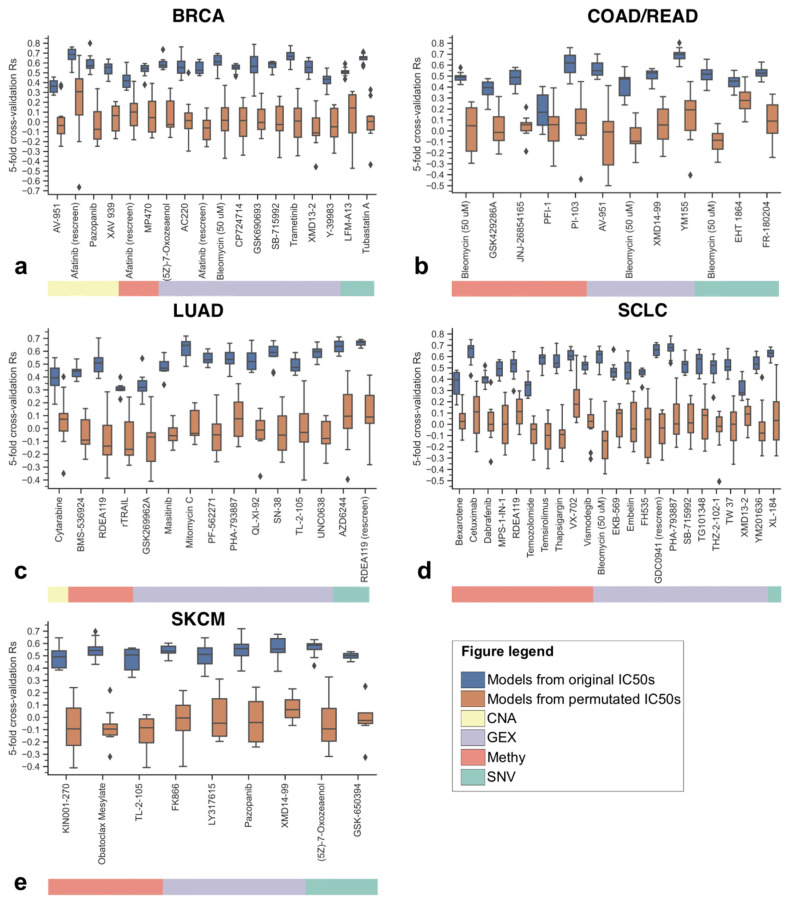
Performances of the 77 predictive XGB-OMC models using real log IC50 values (blue) and their corresponding models using permutated log IC50 values (orange). In each case, performance is calculated as Spearman’s correlation (R_s_) between predicted and observed log IC50 values (the boxplot is formed with the five validation R_s_ values from the five-fold cross-validation using the full dataset for that case). All five cancer types are shown (**a**: BRCA, **b**: COAD/READ, **c**: LUAD, **d**: SCLC, **e**: SKCM), each predictive model identified by its drug (name) and molecular profile (horizontal colour bar below each plot; see legend for correspondence). Of all models, the model for PFI-1 in COAD/READ is by far the worst performer when compared to the permutated log IC50s (*p*-value = 0.27). This model obtains borderline performance on not only the test set (R_s_ of 0.31), but also on the validation set (R_s_ of 0.29). The odds that the other models are being obtained by chance are very unlikely (*p* < 2.2 × 10^−16^, t-test). BRCA: breast invasive carcinoma, COAD/READ: colorectal adenocarcinoma/rectum adenocarcinoma, LUAD: lung adenocarcinoma, SCLC: small cell lung cancer, and SKCM: skin cutaneous melanoma.

To further assess whether these 77 models are truly predictive, we evaluated each selected OMC subset by performing 10 five-fold cross-validation runs on the full dataset (i.e., merging training and test sets for the considered case), in which each run was initialized with a different random seed. We employed the full dataset of each case for this validation in order to use all instances available to that case, instead of validating partially because of using the much smaller test set. Then, we permutated (randomly shuffled) log IC50s across the same data instances and carried out 10 other five-fold cross-validations per OMC subset. In Figure 2, we compared the performances of XGB-OMC models trained on the measured log IC50 values with those from XGB-OMC models trained using case-specific randomly permutated log IC50 values. This y-randomization [29] validation shows what proportion of the model accuracy comes from the signal in the data. As specified before, we only present the best case, i.e., the most predictive combination of drug, cancer type, and omics type. All omics types and cancer types were analyzed separately to be able to compare them.

We can see a substantial difference in Figure 2, even with just 10 cross-validation runs, between the models trained with the unaltered data (blue) and those trained using randomly permutated log IC50s (green). Comparison using a paired t-test for R_s_ from the predictive model and their corresponding permutated variant indicated a highly significant difference between both model types (*p*-value < 2.2 × 10^−16^ across cases), indicating that these model predictions are extremely unlikely to be due to chance. As can be seen in Figure 2, the XGB-OMC models generally outperform models trained on randomly permutated data (note the much higher cross-validated R_s_ values across the 10 runs). This shows that the OMC models capture properties relevant to the drug response. Only one model was found to be slightly problematic when compared to its permutated counterpart (the DNA methylation model for COAD/READ cell lines treated with PFI-1). Compared to most well-predicted cases, the variance in log IC50 for PFI-1 on the relatively low end (0.209 for PFI-1) when compared to the variances for well-predicted drug–cancer combinations in the validation and test sets, such as Thapsigargin in SCLC (0.588), Bleomycin in BRCA (0.653), Mitomycin C in LUAD (0.473), and PI-103 in COAD/READ (0.776). This difference was much less pronounced when compared to the best SKCM case (Pazopanib, log IC50 variance of 0.268).

For all 268 cases that were potentially predictive (R_s_ greater than 0.25 in all validation folds), we compared the models using the OMC with the corresponding models using all features. As previously seen (Figure 2), 77 of the 268 models were predictive in our external test set when using the OMC. Interestingly, we found that 96 models of these 268 were predictive using all features. Therefore, using the XGB-all results in a slightly larger number of predictive models (35.82%) when compared to XGB-OMC (28.73%). Of the 96 XGB-all cases that were predictive, 55 were also predictive using XGB-OMC, thus indicating consistency between both approaches. In 24 of the 55 shared models, XGB-OMC outperformed XGB-all, with the latter seemingly more predictive. However, we found that the average difference in the test set performance between the two was 0.022 in favor of XGB-all, which is not a statistically significant difference (paired t-test; *p* = 0.374). Therefore, we cannot conclude that both methods perform dissimilarly in these 55 cases in common. It is important to note that XGB-OMC resulted in a major reduction of the number of features: 84% of the considered features were on average removed without losing much, if any, predictive performance compared to XGB-all.

**Figure 3:**
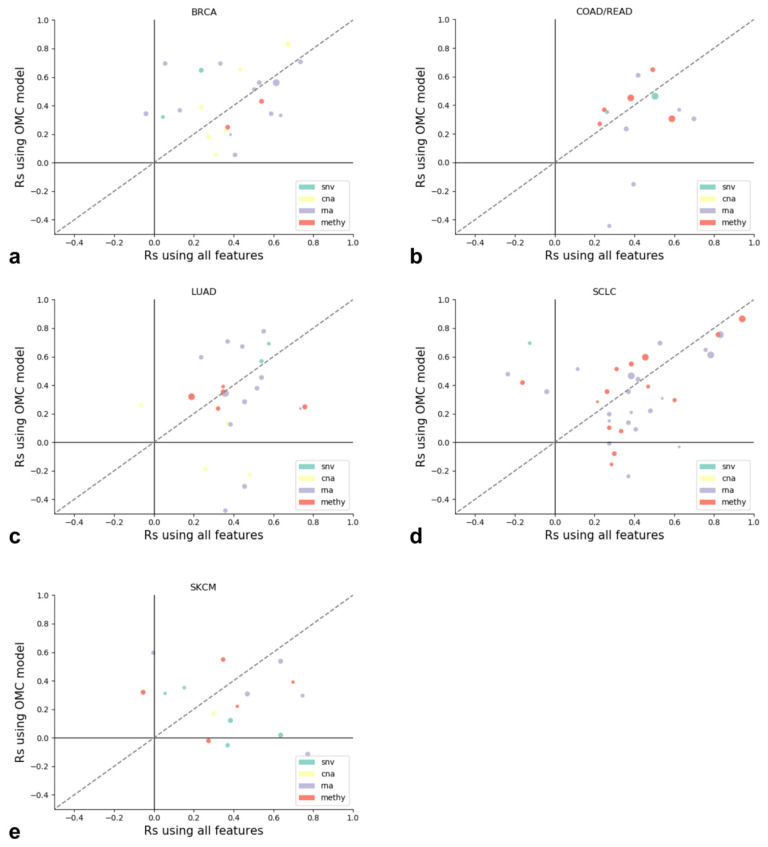
Test set Spearman’s correlations across all 118 cases with predictive models (i.e., R_s_ in validation folds > 0.25 and R_s_ in test set > 0.25). The case with the most predictive omics profile is shown as the node color, while the size of the dots represents the OMC. The dashed gray line represents a 1: 1 ratio between both. In each of the five cancer types (**a**: BRCA, **b**: COAD/READ, **c**: LUAD, **d**: SCLC, **e**: SKCM), the results from both methods are remarkably different overall, despite using the same base learner (XGB) as well as the same set of training and test sets. This strong difference is evidence of their complementarity.

Figure 3 shows a high degree of complementarity between XGB methods. For 41 of the 96 predictive XGB-all models, the XGB-OMC fails to capture potentially predictive characteristics. By contrast, XGB-OMC is predictive in 22 cases where XGB with all features was not. Therefore, the use of both methods is required to obtain predictive models for a larger number of drugs. For those cases below but close to the diagonal, the use the XGB-OMC models is preferred to reduce the feature space with a negligible loss of performance.

From a practical perspective, it is interesting to see that for over half of the cases for which a prediction using the standard all features predictions is possible, these can just as well be described with the OMC subset, as hypothesis testing can then be performed in targeted research experiments, while avoiding the need for costly exome- or genome-wide experiments [30] and qualified personnel that may not be required.

The cases for which XGB with all features did generate less predictive models are also of interest. In each plot in Figure 3, these cases are shown above the dashed diagonal line. Therefore, it is important to consider using the OMC approach as a complement to traditional machine learning, as doing so will add 22 cases that were poorly predicted using the same dataset. In Figure 3, all models that could be considered predictive using either the OMC or the all features method are shown. In total, our study has identified 118 novel XGB models with predictive value (77 predictive with XGB-OMC, complemented by 41 XGB-all), all of which are provided together with their OMC feature set in Supplementary file 1. These 118 feature lists and their corresponding models constitute a large set of complex hypothesis of how cancer cell lines respond to a drug.

Conversely, XGB-all obtained better test set Rs values than XGB-OMC in some cases. One contributing factor could be that the features that correlate the most with IC50s on the training set (i.e., those selected by XGB-OMC) are not as predictive when combined with themselves as they are when combined with features less correlated individually with IC50s. Another factor is that the diversity of cell lines, which can always keep the IC50-correlated features in training cell lines from being sufficiently correlated in test cell lines.

Note that a few XGB-OMC and XGB-all models result in negative correlations with the test set log IC50s in Figure 3. This is likely caused by the small training set sizes. We rely on estimating the top features within folds, while for the final model, we correlate to the full training set. When there are no features that strongly correlate with the log IC50, these feature rankings can be prone to change, resulting in worse performance. The growing availability of drug screening data is expected to mitigate this issue.

### 3.2. Identifying the Most Predictive Molecular Profile for Each Drug and Cancer Type

**Figure 4:**
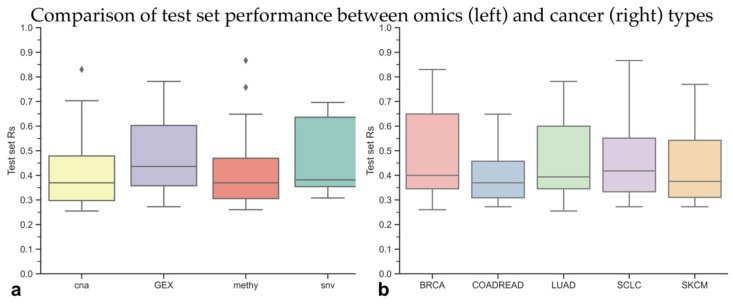
Test set performances of the 118 models identified as predictive in this study. We grouped all the test set performances of the predictive models per omics profile (Panel **a**). Interestingly, there is no universally better omics data type (*p*-values > 0.05, t-test). However, gene expression (GEX) was characterized by high predictive performance, and it resulted in the largest number of predictive models. In panel **b** (right), we visualize the predictive performance of all 118 XGB-OMC and XGB-all models when grouped per cancer type. As in panel a, no cancer type is significantly better predicted than another (*p*-values > 0.05, t-test). Both plots show that predictive models can be built for each omics profile and cancer type.

We observed that the identity of the most predictive profile depends strongly on the considered case. Note that there are more predictive models than drug–cancer type binomials, as some of these cases are well predicted using more than one profile. For example, CNA profiles were employed by only 13 of the 118 predictive models (11 of these 13 were the best model for the case). Surprisingly, we found that only 13 SNV models were predictive (12 of these were the best model for the case). This was in stark contrast to real-valued profiles, which did indeed seem to be much more informative: 60 predictive models using gene expression data (56 best models for the case) and 32 predictive models’ DNA methylation data (31 best model for the case). Although much information for the binary data types (SNV, CNA) may be lost in the feature creation step (e.g., choice of only census genes, disregarding the specific mutation, disregarding the number of copies …), our results based on the GDSC features seem to suggest that for exploratory analysis, gene expression may be the most likely data type to result in predictive models (50.8% of predictive models). Test set performances by means of R_s_ are shown in Figure 4 for both molecular profile types (left) and cancer types (right).

In Figure 4B, we can see the test set performances for models belonging to the different cancer types. These differences are not statistically significant (*p*-values > 0.05, t-test). In total, we established 24 predictive models for BRCA, 16 for COAD/READ, 25 for LUAD, 37 for SCLC, and 16 for SKCM across all data types. In contrast to omics profiles, the distribution of the 118 predictive models across cancer types is much more even, complementing their equivalent overall performance characteristics, which suggest we are not biasing toward a single cancer type. The two cancer types with the largest number of cell lines (LUAD and SCLC) are also those for which the largest number of predictive models was obtained, despite the predictive models from some cancer types coming from a similar number of analyzed cases: LUAD (864), SCLC (647), BRCA (600), COAD/READ (362), and SKCM (694). This further supports the importance of larger datasets. An ANOVA analysis performed in R could not find a statistically significant effect of the cancer type, indicating that no cancer type was better predicted than the rest.

**Figure 5:**
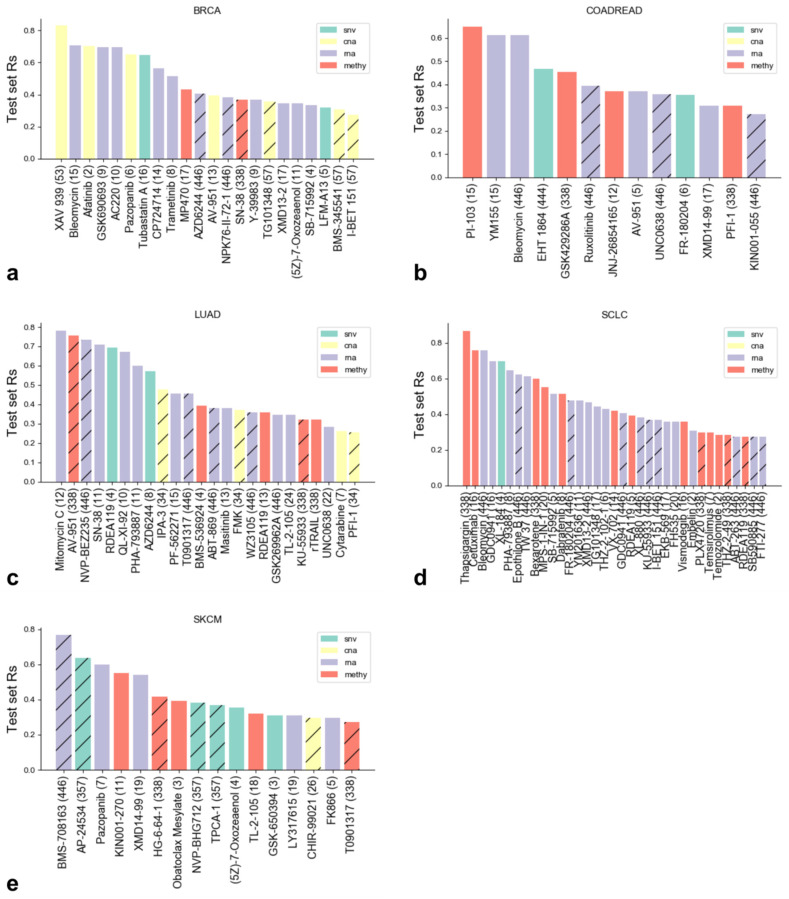
Test set Spearman’s correlation (Rs) between predicted and observed logIC50 of the best cases from each cancer type (**a**: BRCA, **b**: COAD/READ, **c**: LUAD, **d**: SCLC, **e**: SKCM). The data type of the best case (i.e., highest Rs) is shown as color. If the bar is hatched, this indicates that using the XGB-all led to a predictive model where XGB-OMC did not (test set Rs < 0.25). Note that gene expression is the single most informative profile across the considered cancer types. The contributions the other omics data types can vary greatly based on the cancer type.

Figure 5 compiles test R_s_ values between predicted and observed logIC50 of the best cases from each cancer type. In the majority of cancer types, gene expression results in substantially more predictive models than the other three molecular profiles. This is consistent with the highest proportion of predictive models arising from gene expression datasets (60 of the 118 models). However, the relative numbers of predictive models across omics profiles are strongly cancer-specific, as it can be seen in Figure 5. Interestingly, some cases, such as Afatinib and Bleomycin on BRCA, have predictive models with more than one molecular profile (Figure 5 only presents test set performances for the best profile, which are those with the highest test set Rs). This figure also shows the drugs with predictive models on each cancer type (BRCA: 22 drugs, COAD/READ: 13 drugs, SKCM: 16 drugs, LUAD: 24 drugs, SCLC: 35 drugs).

It is noteworthy that of the 12 cases best predicted with CNA features, 7 are found in BRCA. Similarly, SCLC seems to have a proportionally larger presence of drugs that can be predicted with methylation information (13 of the 35 models), while CNA does not seem to play as much of a role as it does in BRCA. On the other hand, SNV seems to be more relevant for SKCM. Although this relation between predictive omics data types and cancer type depends strongly on the drugs tested, extensive reviews have been presented describing the importance of a given data type to specific cancers, such as the role of DNA methylation in SCLC [31].

### 3.3. The Best XGB-OMC Model per Cancer Type as Case Studies

As case studies for discussion, we selected the best XGB-OMC model per cancer type, resulting in five distinct case studies consisting of two gene expression models, two DNA methylation models and a CNA model. These models, together with the test set performance of both corresponding XGB-OMC and XGB-all, are shown in Table 1. The R^2^ metric (coefficient of determination), which is an alternative parameter to gauge model performance, is also shown. Two models use all features, while the other three require far fewer features to be predictive, as indicated by the OMC column. Note that the Pazopanib-SKCM could not be predicted at all using all 450 GEX features, unlike when using the OMC to build XGB models with just seven of them.

We highlighted the best XGB-OMC model per cancer type to discuss in detail (Table 1). The first of these five case studies is the DNA methylation model to predict the response of SCLC cell lines to Thapsigargin. The plant-derived compound Thapsigargin is a Ca^2+^-ATPase antagonist that can increase intracellular Ca^2+^ concentrations in small cell lung cancer cells and thus has an effect on excreted autocrine growth factors, such as serotonin [32], which is represented at the gene expression level [33]. Furthermore, SCLC shows very distinct methylation patterns that strongly correlate with E2F (E2F transcription factor 1) expression, which is a cell cycle regulator for the transition from G1 to S phase. The activation of EZH2 (enhancer of zeste 2 polycomb repressive complex 2 subunit) has been shown to be a direct consequence of RB1/E2F pathway dysregulation in SCLC [34]. EZH2 is a histone methyltransferase, with EZH2 inhibiting tumor growth in PDX (Patient-Derived Xenograft) models [31]. Our results, based on cell lines, are hence consistent with in vitro and in vivo findings found in the literature, which indicate that the RB1/E2F pathway, and consequently this DNA methylation pattern, may hold predictive value for SCLC treatment strategies.

The second case study is the copy-number alteration model to predict the response of BRCA cell lines to XAV-939. XAV-939 inhibits tankyrase 1 and 2, thus inhibiting Wnt/β-catenin-mediated transcription and stabilizing the cytoplasmic axin levels. Genes associated with the Wnt pathway have high expression in triple-negative breast cancer [35], which makes it an interesting therapeutic prospect to fight this cancer type, representing between 10% and 20% of all breast cancer cases. In our feature set, we found all 53 BRCA-specific CNA to be required for the optimal XGB model (24 were amplifications and 29 constituted deletions). We mapped the CNA-altered region to the gene identifiers of the contained genes with the GDSC-provided files and performed an overrepresentation analysis with pathway terms. Enrichment analysis using KEGG annotation indicated a slight enrichment of BRCA (*p* = 0.029, ID: hsa05224), thanks to the presence of APC2 (APC regulator of Wnt signaling pathway 2), a Wnt signaling pathway regulator, E2F3 (E2F transcription factor 3, also involved in Wnt/β-catenin regulation), WNT5B (Wnt family member 5), EGFR (epidermal growth factor), and a large number of fibroblast growth factors being present. The presence of several Wnt signaling-related features in the dataset and the model’s performance in a cancer type in which the drug has been found to have an effect increases confidence in the polygenic complex hypothesis embodied by the XGB-OMC model.

The third case is the gene expression model to predict the response of LUAD cell lines to Mitomycin, which again seems to be in a clinically relevant tissue for the drug. Thus far, 11 clinical trials evaluate Mitomycin to treat lung cancers (clinicaltrials.gov). In fact, this drug is already indicated for certain lung cancer types (Drugbank ID: DB00305), e.g., it has been used since 1984 for non-small cell lung cancers [36]. Mitomycin is an antibiotic produced by amongst others the actinobacteria *Streptomyces caespitosus*. Our OMC subset contained 12 gene expression features: NCOR2_expr, MCM3_expr, AMOT_expr, POLR2B_expr, ARFGEF2_expr, U2AF1_expr, NDRG1_expr, MYD88_expr, NTRK2_expr, ATF1_expr, SHMT1_expr, and HNRPDL_expr. Interestingly, MYD88 (MYD88 innate immune signal transduction adaptor) works in close relation with members of the TNF (tumor necrosis factor) family [37], which has been frequently linked to Mitomycin and its Fas/FasL (Fas cell surface death receptor)-dependent apoptosis in cervical carcinoma [38]. Furthermore, several of the features in the OMC set are associated at the protein level with NQO1 (NAD(P)H quinone dehydrogenase 1), which has been described as having an effect on the sensitivity to Mitomycin C [39]. Finally, protein levels of HNRP (heterogeneous nuclear ribonucleoprotein) proteins, one of which is in the OMC set, are known to rise after DNA damage [40], such as the damage caused by oxidative stress induced by Mitomycin.

The fourth case study is a DNA methylation model that is used to predict the response of COAD/READ cell lines to PI-103. PI-103 is an inhibitor of PI3K (phospho-Inoside 3-kinase) and mTOR. Previous research indicated that the effects of PI-103, such as the downregulation of choline kinase α, were observable in prostate and colon carcinoma cell lines using magnetic resonance spectroscopy [41]. PI-103 is known to enhance radiosensitivity in several cell lines (including colorectal) and reduced phosphorylation of AKT1 (AKT serine/threonine Kinase 1) at serine 473 [42]. In addition, PI-103 has also been found to slow down tumor growth in PDX models [43]. Hence, our models identify again a predictive case with literature and in vivo experimental support. Since PI3K is has a downregulatory effect of the PI3K pathway, we expected some members of our OMC subset to be related to this pathway. We found that a complexity of 15 CpG islands (iCpGs) [5] best describes the drug response to PI-103. Interestingly, several of these were also present in other cancer types in which the effect of PI-103 has been described. We used the GDSC-provided file (TableS2H.xlsx) to obtain the names of the genes regulated by the considered iCpGs. This resulted in the following set of 18 genes: RAPGEFL1, RRS1, ADHFE1, LIPG, OXR1, ADPRH, ENC1, GSTT1, HSD11B2, ZNF3, DNAH10, PAQR8, SIAH1, HOXA11AS, HOXA11, KAT2A, HSPB9, and P4HB. P4HB (Prolyl 4-hydroxylase subunit beta) is closely associated with AKT1, which is known to be affected by PI-103 [42]. Moreover, GSTT1 (Glutathione S-transferase (GST) theta 1), HOXA11 (Homeobox A11), and SIAH1 (Siah E3 ubiquitin protein ligase 1) are associated with TP53 (Tumor protein P53) in the StringDB [44] network, the latter of which was shown to be affected by PI-103 in AML [45] and is closely associated with AKT1. These studies further support that the methylation status of the promoter regions of at least some OMC-selected genes are highly relevant for PI-103′s drug response.

The last case study is the gene expression model to predict the response of SKCM cell lines to Pazopanib. Pazopanib is a tyrosine kinase inhibitor with antineoplastic activity. It has been used to treat renal cell cancer and soft tissue sarcoma. The drug acts against vascular endothelial growth factors 1, 2, and 3, as well as platelet-derived growth factor receptor beta [46]. We found that an OMC of 7 was sufficient for a gene-expression model to be predictive. Our features were TRERF1_expr, KDR_expr, VHL_expr, PIP5K1A_expr, FMR1_expr, PTPRU_expr, and EPHA4_expr. None of these are a known target of Pazopanib. However, we again found close associations between members of our OMC subset with the known drug targets. For example, PTPRU (Protein tyrosine phosphatase receptor type U) is a direct neighbor of KIT (KIT proto-oncogene, receptor tyrosine kinase), with a combined string score of 0.947, indicating very high confidence. KDR (kinase insert domain receptor) associates with the known targets PDGFB (platelet-derived growth factor subunit B, 0.559), FLT1 (vascular endothelial growth factor receptor 1, 0.963) and VEGFR3 (vascular endothelial growth factor receptor 3, 0.943). VHL (Von Hippel-Lindau syndrome), also in our OMC set, associates with both KDR and FLT1. Other than finding immediate connections in a protein–protein association network, we could find that a clinical trial involving Pazopanib for stage IV cutaneous melanoma was completed in 2010 and reported one patient of the 13 tested with a partial or complete response (https://clinicaltrials.gov/ct2/show/results/NCT00861913). It was also further tested in combination with paclitaxel for advanced melanoma, with the authors describing the potential as interesting [47]. Lastly, Pazopanib also had a reported activity in melanoma xenografts [48]. Overall, these observations again suggest that the predicted indication may be clinically relevant.

## 4. Methods

### 4.1. GDSC

All datasets used were made publicly available by the authors of the 2016 GDSC study through their publication-specific download portal, which can be found at http://www.cancerrxgene.org/gdsc1000/GDSC1000_WebResources/Home.html. This page provides several Excel sheets referred to in the Iorio et al. paper [5] as Supplementary Materials. All datasets containing cell line phenotypic data, drug response, and information regarding primary tumors used in this study were downloaded through this web portal. The pre-processed datasets were used, as the steps taken by Iorio et al. [5] result in a well-annotated list of features with known functional role in cancer.

### 4.2. Cell Line Information (Metadata)

Throughout the GDSC, both the actual name of the cell line, as well as its COSMIC identifier were used. We created a simple mapping between these two identifiers using the Excel sheet named TableS1E.xlsx (tab “TableS1E-CellLines”). This file also contained further information, such as GDSC tissue type, growth medium, growth properties, microsatellite instability, and TCGA abbreviation. For each cell line, we only retained the identifiers (COSMIC identifier, sample name) and the TCGA abbreviation of the corresponding cancer type.

### 4.3. Drug Response (Continuous Variable)

Drug responses were provided as the natural logarithm of half the maximal inhibitory concentration in a data matrix (TableS4A.xlsx, tab “TableS4A-IC50s”), which we subsequently converted to logarithms with base 10. Cell line information was then merged with the drug response and other phenotypic properties (genomics, transcriptomics, methylomics). Pre-processing steps for the four molecular profile types (SNV, CNA, gene expression, methylation) are further described below.

### 4.4. Single-Nucleotide Variants (SNV, Binary Feature)

Using whole-exome sequencing data for 48 studies with matched tumor-normal samples, Iorio et al. [5] combined the outputs of three variant calling algorithms to predict 470 important cancer genes in primary tumors (TableS2A.xlsx, tab “TableS2A-CancerGenes”). In TableS2B.xlsx (tab “TableS2B-TumourVariants”), which is also available from the download portal, they cover the mutations of all 18,406 genes in primary tumors, while “TableS2C.xlsx” (tab “TableS2C-CellLineVariants”) describes 486,243 variants and their classification (missense, nonsense, frameshift, ess_splice, inframe, stop_lost, gene_fusion) found in 19,100 genes for the 1001 GDSC cell lines in 30 cancer types. A total of 470 gene names appeared in the primary tumor list (“TableS2B.xlsx), while these 470 predicted cancer driver genes (“TableS2A.xlsx”) were also present in the cell line variant list (“TableS2C.xlsx”). The presence of mutations in these 470 cancer genes in both cell lines and primary tumors signifies their potential clinical relevance. We assigned a 1 to all cell lines in which the feature (gene) carried a mutation, while zeroes indicated wild-type status. This resulted in the 470 SNV features employed.

Iorio et al. [5] further performed a driver mutation analysis with 358 driver mutations reported in primary tumors and 310 in cell lines (293 overlapping between both, available as BEM (binary event matrix) files from the download portal). This constitutes a substantial reduction in feature space. We did not make this distinction between driver and passenger mutations, as the relation between the state of a gene mutation (driver, passenger) and the actual drug response is currently not well understood. Therefore, we opted to err on the side of caution and relied on the dataset itself to identify lists of features with an effect on predictive performance.

### 4.5. Copy Number Alterations (CNA, Binary Feature)

CNA data for cell lines were obtained from TableS2G.xlsx (tab “TableS2G-CellLinesRACSs_CNA”). CNAs were identified by Iorio et al. [5] through ADMIRE [49] analysis over 27 cancer types. Note that no CNA data were released for SCLC. From TableS2G.xlsx, we extracted all segments listed under the column header “Region identifier”. These segments were annotated with the prefix cna, the cancer type, and a number (e.g., cnaLUAD1). Contained genes are also indicated between brackets where applicable (e.g., cnaLUAD35 (MECOM)). In total, we obtained 558 unique segments for cell lines. From TableS2F.xlsx, which contains the altered segments in primary tumors, we again filtered out everything but the contents of the “Region identifier” column. This resulted in 851 altered segments for primary tumors. We again selected the intersection between the data for the cell lines and the primary tumors as the section being most relevant for further studies in patients. All 558 segments in the cell lines appeared in primary tumors, while the other 293 were unique to the primary tumors, as reported in the original article [5]. Similar to the genes carrying SNVs, we opted to indicate a copy number altered state as a binary value (either 1 if True or 0 if not). We used all 558 segments that were not unique to cell lines. These are cancer-specific, and hence, each cancer type employed a different set of CNA features.

### 4.6. Transcriptome (GEX, Continuous Feature)

RMA-normalized pre-processed expression information (sanger1018_brainarray_ensemblgene_rma.txt) was also available throughout the article download portal. However, in this case, it links out to the main GDSC portal. We used the latest dataset version of 2 March 2017. The file contains the Ensembl gene identifiers for the genes and COSMIC identifiers for the cell lines. Out of the 1018 cell lines in the file, we retained only those with a COSMIC identifier part of the 1001 cell lines presented in the original GDSC study [5]. The list of genes we reduced from the 17,737 Ensembl gene identifiers to the list of 466 genes to represent the information also covered in SNV. To this end, we mapped the identifiers to the HGNC gene name using the mapping files provided by Ensembl BioMart [50] (Supplementary file 2). A total of 446 out of the 470 HGNC gene symbols could be successfully mapped to their corresponding Ensembl identifiers. The 24 missing entries could all be attributed to use of a secondary name or lack of presence on the array, which we corrected accordingly. Subsequently, 450 features had both expression and mutation information available, and these were used as gene expression features. We retained all gene expression values as continuous variables in order to keep all information content of the data type. Therefore, each cell line was characterized by 450 GEX features.

### 4.7. Methylome (Methy, Continuous Feature)

The list of informative CpG islands (iCpGs) hypermethylated in primary tumors (“Table S2I.xlsx”, GDSC article-specific download portal) contained 357 unique sites in the column “HyperMethylated iCpG”. We further downloaded the iCpGs found in cell lines by Iorio et al. [5] (TableS2J.xlsx), which contained 338 unique hypermethylated iCpGs and created the intersection between both lists for clinical relevance. All 338 iCpGs found in cell lines occurred in the primary tumors. As the average pre-processed β-values for each of these sites were also available (“F2_METH_CELL_Data.txt”), we opted to keep the content type as rich as possible and use methylation as a continuous feature based on the average β-values per site. We created the methylation data matrix with 338 iCpGs as features and cell lines as index using the mapping file “methSampleId_2_cosmicIds.xlsx”, which is also available from the GDSC study [5].

### 4.8. Generation of Non-Overlapping Training and Test Sets

Starting from the pre-processed data, we compiled a dataset for each cancer type with at least 45 treated cell lines annotated with response to a given drug. This modeling choice takes as reference the lowest training set size successfully used in the literature [11], where 28 drugs with 35 profiled cell lines were used as a training set. To be on the safe side, we only considered cases with at least 35 training cell lines. Since we also required an external test set of at least 10 cell lines, only cancer types with at least 45 cell lines in the GDSC [3] were analyzed.

Per case (i.e., a combination of drug, cancer type, and omics data type), an external test containing 10 cell lines was created. Since we intended to create a test set representative of the full drug response range, we ranked all log IC50 values in ascending order and placed the second most extreme values (i.e., the log IC50 just below the maximal and that just above the minimal log IC50 for the considered case) to the external test set. The eight other test set cell lines were selected in an equidistant manner based on the rank of their log IC50 value. This stratification procedure covers a representative range of the drug response and avoids the situation that may appear with fully random selection in which either all high or all low log IC50s are selected, resulting in a biased test set. These 10 cell lines were excluded from the model training set and were therefore not used at all for model building or selection. Thus, the extreme IC50 values are always part of the training set.

### 4.9. Optimal Model Complexity Estimation

XGB will also be employed in conjunction with a strategy to identify the Optimal Model Complexity (OMC) for each case (i.e., each trinomial drug-cancer type-omics profile). In our OMC workflow and for each case, we first rank features based on their correlation with drug response and then train the k^th^ model with the top k features. The idea is to discard the many thousands of uninformative features by selecting the model trained only with those with the highest information content. Thus, the optimal k (k_opt), and a fast approximation to the optimal subset of features of size k_opt corresponds to the model with the highest cross-validated performance in a given case. Results for all cases are reported on test sets that were neither used for training nor for model selection.

### 4.10. Model Selection by Stratified Five-Fold Cross-Validation on the Training Set

Five-fold cross-validation was used as strategy to select the OMC. For our five-fold cross-validation on the training set, we iteratively generated an internal training set (80% of *n* cell lines in the training set) used to build the model and a validation set (remaining 20% of *n* cell lines in the training set) to internally test. This ensured that our internal validation sets were of similar size to the external test set. To further guarantee the representativeness of the external test set, we used the same stratification procedure described in “Generation of non-overlapping training and test sets”. Per fold and per possible complexity between 2 and *n*/2, the correlation between predicted and observed log IC50 values was calculated using the Spearman correlation. Complexities that failed to provide to outperform the minimal Rs threshold of 0.25 in any of the five validation folds were discarded. We also rejected models that predicted a constant output value regardless of input data. The enforcement of these rules slightly penalized lower complexities in scenarios where the response range (distance between minimal and maximal log IC50) was low and the models predicted the same drug response irrespective of the biological features used input. The Optimal Model Complexity (OMC) was chosen as the complexity for which the median Spearman correlation across the five folds was highest. As such, it is the number of input features that results in the best median model performance across the five validation folds.

### 4.11. Pre-Ranking Feature Input

The OMC strategy starts by first ranking the input features based on the strength of their association with the log IC50 for a particular case. For discrete inputs (SNV, CNA), this strength is calculated as the *p*-value of R’s [51] wilcox.test function, as the corresponding Python implementation uses approximations. R objects were imported in Python using rpy2 (https://rpy2.bitbucket.io). *p*-values for continuous input were calculated using a Spearman rank-correlation test [51].

### 4.12. Obtaining the OMC for a Given Drug Fold

After ranking the features from likely to be most relevant (lowest *p*-value) to increasingly less significant, we defined that the optimal complexity was between 2 features and *n*/2 features, where *n* equals the number of cell lines available in the training fold for a particular case. The upper threshold (*n*/2 features) was chosen to guarantee that we have at least two data instances per feature. In combination with the feature reduction by OMC, the resulting models should be less prone to overfitting. We also trained models using the maximal possible complexity (i.e., all features), as some cases may require the combination of more features for optimal performance.

### 4.13. XGBoost (XGB)

We downloaded the code for XGB (https://github.com/dmlc/xgboost). We first trained and tested all models using default parameters and all features to investigate if XGB captured some of the underlying biology and performed better than random. For the actual model complexity estimation, we opted to use a more conservative learning rate (0.05) than the default value of the implementation, a maximal tree depth of 6, and a larger number of trees (700), as described by Sheridan and co-workers [24]. Subsampling of row (cell line) and column (feature) features per boosting iteration without replacement was set as 0.8 for both in the OMC estimation model. The 80% denotes the fraction of features and observations that was used to build a tree. A case-specific comprehensive grid search for optimal hyperparameter values is expected to improve results further, but it is impractical as the number of cases is too large.

### 4.14. Final Model Performance Estimation

For the construction of the final models, the full training set was used for model training. First, features were pre-ranked as described earlier. Second, the top OMC features were used as input for the models, which were again trained based on 10 random seeds. All other parameters were kept the same as in the random seed five-fold CV loop (settings as in the conservative approach presented by Sheridan et al. [24]). Finally, model performance was calculated using the Spearman metric between the 10 predicted log IC50s and the corresponding test set values observed in the 10 cell lines of the external test set.

### 4.15. Pathway Enrichment Analysis

Enrichment analyses were performed using the Webgestalt tool [52] for the exploratory analyses for the case studies. A minimal significance of 0.05 with Benjamini–Hochberg correction over 1000 permutations for *p*-value estimation was set. The minimal threshold of features required in a category for a category to be recognized as potentially interesting was defined as 5 (default tool setting). Only annotations for *Homo sapiens* were used for the GOSlim [53] and KEGG [54] pathway sets.

### 4.16. Statistical Tests

Statistical tests were executed in R [51], using Python in combination with the rpy2 interface (http://rpy.sourceforge.net/rpy2).

## 5. Conclusions

Cell lines have demonstrated to be highly useful cancer models, especially in a pharmacogenomics context. They are cost-efficient, quick to grow, and amenable to high-throughput experiments [55]. However, similar to all disease models, they also suffer from several inherent limitations. For example, intra-tumor heterogeneity, extracellular environment, and immune system response are not modeled in cell lines, which may lead to alternative disease models, such as PDX [56], being more predictive. Cell lines are furthermore prone to divergence across passages [51]. Despite these issues, several cell lines have proven to be relevant by retaining sufficient correlation to primary tumors [55,57,58]. Given their suitability for high-throughput experiments [49], they are also the model for which most phenotypic and pharmacological information is publicly available. For many drugs, only much smaller amounts of pharmacogenomics datasets, if any, has been released using more patient-relevant in vivo models [59,60,61,62]. Consequently, often the only data that can be used to predict drug sensitivity comes from cell lines, thus having their niche in guiding precision oncology efforts [55].

Our results indicate that XGB, combined with a strategy searching for the Optimal Model Complexity (OMC), can generate very predictive models using only concise sets of tumor features. The latter means that only a fraction of the initially considered features need to be determined in additional cell lines, which is far less resource-intensive. We further demonstrate that the predictive models are very unlikely to arise by chance (Figure 2). In addition, they are largely complementary to those generated by XGB using all features (Figure 3). Predictive models were found in each cancer type and with every molecular profile (Figure 4). On average, no omics profile or cancer type obtained models with higher predictive accuracy than the rest. However, within a given cancer type, some molecular profiles were overrepresented among predictive models (Figure 5). For instance, CNA profiles were predictive in BRCA cell lines, but not in SCLC cell lines where GEX and DNA methylation profiles were the most predictive. Overall, we analyzed a total of 792 drug–cancer type binomials. Figure 5 shows that XGB-OMC achieved predictive models for 110 of these 792, with XGB-all finding models for 37 binomials more. This evidences how challenging drug response prediction on cancer cell lines is, as discussed elsewhere [63,64,65].

Although we demonstrate the advantages of the approach using only the best-performing models for each cancer type, many more interesting cases were also found (see Figure 5). For example, some cases recapitulated the role of KRAS and BRAF, who are extensively used as clinical biomarkers [66]. In LUAD, the mutation of KRAS appears by estimation in 17% of all cases, while only 2% carries a BRAF mutation [66]. Given all the evidence outlined in their review, the authors suggest screening BRAF, KRAS, NF1, and gain information on the effective use on anti-EGFR treatment. KRAS was suggested as a biomarker for treatment with AZD6244 (Selumetinib), which is a MEK1 inhibitor [67], while another phase II clinical study identified that NF1 was also indicative of tumor response to AZD6244 [68]. Our results support the use of mutations in both KRAS and NF1 in combination with those in the other selected genes (KRAS_mut MYH14_mut FN1_mut MAGI2_mut CHD9_mut ARHGEF6_mut NF1_mut EFTUD2_mut) as a potentially viable biomarker set for AZD6244 response. This set is much richer than predictions involving only KRAS and/or NF1.

Given that all of the five case studies were found to be in relevant cancer types and showed tight integration with the verified or suggested drug mode of action, we believe that the remaining 113 predictive models, as well as the OMC methodology, could also provide useful starting points for researchers investigating how the response of these drugs are affected by the molecular background of tumors. From a methodology research perspective, we have focused on investigating the integration of the OMC strategy with a state-of-the-art embedded-feature-selection algorithm (XGBoost) for supervised learning. With this purpose, we have performed comprehensive comparisons across a range of problem instances of both XGB-OMC and XGB-all (i.e., XGBoost as downloaded without OMC). The comparisons have been discussed in a non-competitive manner because the results showed that using both algorithms is better than using either of them alone. In the future, we would like to look at the related question of whether integrating OMC with other machine learning algorithms is also beneficial. However, such large-scale systematic benchmarking exercise requires a dedicated study to properly discuss the many results that would be obtained.

## Figures and Tables

**Table 1:** The most predictive XGB-OMC models per cancer type. Optimal complexity, test set performance (Spearman), and the test set performance (Spearman) of the corresponding all features models are shown (BRCA: breast invasive carcinoma, COAD/READ: colon adenocarcinoma/rectum adenocarcinoma, LUAD: lung adenocarcinoma, SCLC: small cell lung cancer, SKCM: skin cutaneous melanoma). For each of these models, validation set performance also exceeded Rs > 0.25 in all validation folds.

Profile	Cancer Type	Drug Name	OMC	Test R_s (XGB-OMC)_	Test R_s (XGB-all)_	Test R^2^_(XGB-OMC)_	Test R^2^_(XGB-all)_
Methy	SCLC	Thapsigargin	338 (all)	0.867	0.939	0.535	0.609
CNA	BRCA	XAV 939	53 (all)	0.830	0.673	0.347	0.382
GEX	LUAD	Mitomycin C	12	0.782	0.552	0.465	0.280
Methy	COAD/READ	PI-103	15	0.648	0.491	0.395	0.296
GEX	SKCM	Pazopanib	7	0.600	−0.006	0.321	−0.231

## Supplementary Materials

The following are available online at https://www.mdpi.com/2218-273X/10/6/963/s1, Code supporting the conclusions of this paper and its instructions of use are available at http://ballester.marseille.inserm.fr/GDSC_XGB-OMC.zip. The instructions also specify which input data files are used and hence have to be downloaded from https://www.cancerrxgene.org/ XGB models along with their predictive accuracies and OMC feature sets are specified in Supplementary File 1, Correspondence between Ensembl identifiers and HGNC gene names as provided by Ensembl BioMart in Supplementary file 2.

## Abbreviations

CCLE	Cancer Cell Line Encyclopedia
CTRP	Cancer Therapeutics Response Portal
GDSC	Genomics of Drug Sensitivity in Cancer
GO BP	Gene Ontology – Biological Process
OMC	Optimal Model Complexity
PDX	Patient-Derived Xenografts
RF	Random Forest
XGB	Extreme Gradient Boosting
